# Spatial distribution patterns and causal mechanisms of rainfall-induced clustered landslides: A case study from Southwestern Yunnan Province, China

**DOI:** 10.1371/journal.pone.0347603

**Published:** 2026-04-28

**Authors:** Zongheng Xu, Yun Zeng, Yilong Li, Mei Yang

**Affiliations:** 1 Department of Geography, Yunnan Normal University, Kunming, Yunnan, China; 2 Key Laboratory of Plateau Geographic Processes and Environment Change of Yunnan Province, Kunming, Yunnan, China; Auckland University of Technology, NEW ZEALAND

## Abstract

Based on two major rainfall events that triggered clustered landslides in Changning, Baoshan, southwestern Yunnan Province, China, this study integrates multi-scale spatial analysis, systematic field investigations, and geomechanical characterization to examine the distribution patterns and rainfall-triggering mechanisms of clustered landslides. A total of 255 landslides were identified, exhibiting a belt-like distribution along the main river valleys with pronounced clustering at mid-to-high elevations and moderate slopes. High-incidence zones are concentrated at elevations of 1860 ~ 1900 m, slopes of 20° ~ 40°, and outcrops of gray-black thin-layered microcrystalline schist and gray-white quartz schist, with a strong orientation preference toward south- to southeast-facing slopes. Notably, landslide density on natural slopes located more than 500 m from roads is higher than in adjacent areas, indicating that the spatial pattern is primarily controlled by topography-lithology combinations rather than anthropogenic engineering disturbances. Microstructural analysis reveals pervasive schistosity, cleavage fractures, and clay-rich weathering bands, with weathering driving the evolution from dense to porous, loose structures, forming highly permeable, low-strength weak zones. Direct shear tests further show significantly reduced shear strength in shallow layers and weakened zones, which are highly sensitive to water content and external disturbances. Intense rainfall rapidly infiltrates, increases pore water pressure, and softens shallow layers, activating schistosity planes and inducing displacement, thereby promoting clustered and concentrated landslides. This study clarifies the spatial controls and coupled internal–external mechanisms of rainfall-induced landslides in metamorphic rock regions, providing essential scientific guidance for landslide hazard assessment and disaster mitigation planning in Yunnan’s mountainous watersheds.

## 1. Introduction

Intense rainfall is widely recognized as the primary trigger of shallow landslides and debris flows in mountainous regions, often exhibiting sudden, concentrated, and clustered behaviors in both space and time [1]. Understanding the spatial distribution patterns and triggering mechanisms of rainfall-induced landslides is crucial for elucidating the predisposing-to-triggered processes and for implementing effective hazard mitigation and risk management strategies [2]. Such studies provide essential insights into the coupling between topography, lithology, weathering, and hydrological processes, while also informing the identification of landslide-prone areas, regional susceptibility assessments, and early warning systems [3–5]. In the complex mountainous terrain of southwestern China, intense tectonic activity and intricate geomorphic evolution result in pronounced spatial clustering and multi-factor coupling of rainfall-triggered landslides, underscoring the theoretical and practical importance of investigating their distribution patterns and causative mechanisms [6].

The analysis of spatial patterns is a foundational aspect of landslide hazard assessment and risk management, as it reflects the complex interactions among multiple controlling factors across space and time. Accumulated evidence indicates that the spatiotemporal characteristics of rainfall critically control both landslide initiation and the development of spatial patterns [7–9]. Among these factors, extreme and prolonged rainfall events are generally considered the most influential triggers [10,11]), while the spatial heterogeneity of precipitation can strongly affect the localized response and propagation of shallow landslides at the watershed scale [8]. Beyond rainfall, landslide occurrence is jointly modulated by topography, lithology, land use, and anthropogenic activities. For instance, slope gradient, elevation, aspect, lithology, hydrological conditions, and transportation infrastructure have all been shown to exert significant control over landslide distribution, with slope steepness and proximity to streams often acting as primary determinants [12]. The influence of hillslope curvature and convergence/divergence on shallow landslide initiation has also been increasingly recognized [13,14]. Geological features, including lithology, fault networks, and regional structural conditions, define the intrinsic susceptibility of slopes and serve as critical inputs for numerical modeling [15–18]. Land use and vegetation cover influence landslide probability by regulating soil moisture, surface runoff, and root reinforcement, while vegetation recovery can play a pivotal role in long-term slope stabilization [19]. Anthropogenic activities, such as urban expansion, deforestation, and infrastructure construction, further exacerbate surface disturbances and elevate landslide risk [20,21]. The interplay between seismic events and rainfall is also increasingly recognized as a key factor in landslide susceptibility assessments [5,6,22]. Meanwhile, recent advances in remote sensing, geographic information systems (GIS), statistical modeling, physical simulation, and machine learning have provided effective and powerful tools for analyzing spatiotemporal patterns and their controlling factors [23–25]. Recent research trends further emphasize the multi-scale coupling of hydrological, geological, and topographic factors rather than relying on single-variable analyses. In recent years, research has increasingly shifted from single-variable analyses toward multi-factor approaches, which enable a more comprehensive understanding of landslide spatial patterns and their controlling mechanisms. Empirical investigations based on rainfall–duration (I–D) thresholds have been refined with additional variables, including soil moisture and cumulative rainfall, enhancing the reliability and applicability of early warning systems [26–28] Concurrently, laboratory experiments, field monitoring, and numerical simulations increasingly incorporate unsaturated flow, fracture flow, and strength degradation mechanisms, enabling realistic modeling of the full process from rainfall infiltration and pore-water pressure buildup to mechanical failure, thereby providing deeper insight into the physical triggers of rainfall-induced landslides [25,28–30].

The high-mountain canyon regions of southwestern Yunnan, China, are particularly prone to clustered shallow landslides. However, systematic investigations of their spatial distribution patterns and the dominant environmental controls remain limited, particularly regarding the interactions between geological material properties and rainfall-triggered processes. To address these gaps, this study focuses on clustered shallow landslides induced by two extreme rainfall events in Changning County, Yunnan, during 2015–2016. Using landslide clusters as the primary dataset, the research systematically evaluates spatial distribution patterns and associated environmental factors, including geology, topography, and vegetation, to elucidate the controlling mechanisms of rainfall-triggered landslides. In addition, by incorporating microstructural analysis of rock masses, the study investigates the interaction between geological material properties and hydrological processes, providing a mechanistic understanding of the evolution from predisposing conditions to triggering in clustered landslides. The findings contribute to improved scientific understanding and methodological approaches for landslide hazard assessment and disaster mitigation in high-mountain watersheds of southwestern Yunnan.

## 2. Materials and methods

### 2.1. Study area.

**Fig 1:**
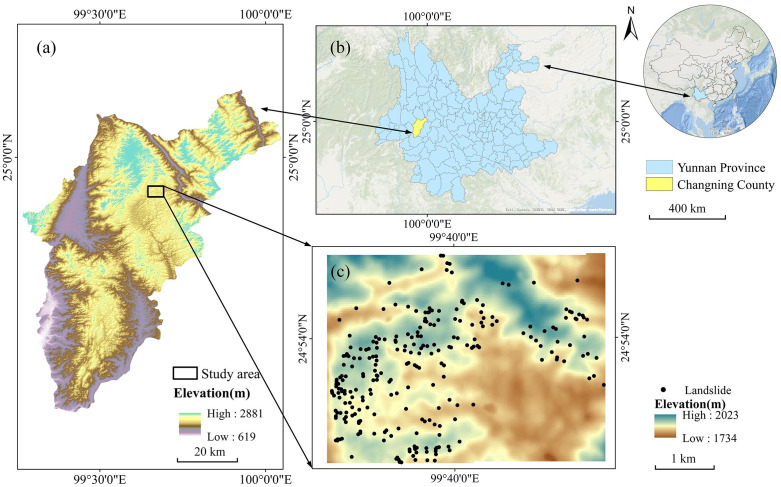
Geographic location of the study area: (a) Changning County, Yunnan Province; (b) location of the study site; (c) spatial distribution of landslides within the study area. (a) and (c) use DEM data provided by the Geospatial Data Cloud, Computer Network Information Center, Chinese Academy of Sciences (http://www.gscloud.cn). (b) uses boundary vector data and basemap services from Map World (National Platform for Common GeoSpatial Information Services) (www.tianditu.gov.cn).

The study area is located in Changning County, Baoshan City, southwestern Yunnan Province, China, between 24°52′49″ ~ 24°54′48″ N and 99°38′42″ ~ 99°41′34″ E, on the southwestern outskirts of Mangshui Town. The spatial extent of the study site spans approximately 4.814 km from east (Xiaolangba) to west (Hexi Reservoir) and 3.678 km from north (Zhongshan area) to south (Baifen area), covering a total area of 17.7 km² (Fig 1a–c). The terrain is predominantly mountainous, characterized by higher elevations in the northwest and lower elevations in the southeast, with a maximum altitude of 2023 m, a minimum of 1734 m, and an average elevation of 1877.3 m. The climate is subtropical humid, with mild temperatures and abundant precipitation, showing clear wet and dry seasons. Observational records indicate a mean annual temperature of 14.9 °C and an average annual precipitation of approximately 1259 mm.

Geologically, Changning is situated in the southern segment of the Hengduan Mountains in southwestern China, representing a key microcontinental block within the eastern Tethyan orogenic belt, and forming part of the Indo-Myanmar tectonic subduction zone [31–33]. The study area, near the Lancangjiang Fault Zone, has undergone multiple tectonic phases, resulting in widespread folding, faulting, and complex bedrock distribution controlled by major faults such as the Changning–Yingpan and Kejie Faults [32]. Crustal heterogeneity within the upper 0–7 km reflects ongoing tectonic activity [34,35]. These processes have shaped a diverse landscape of ridges, basins, and river valleys with pronounced relief typical of southwestern mountainous terrain [31,36]. The combination of steep slopes and complex geological conditions, particularly the widespread distribution of schist, underpins the region’s high susceptibility to geological hazards [31,37], with landslides, debris flows, and slope failures representing the most prevalent hazard types, particularly under conditions of intense rainfall.

**Fig 2:**
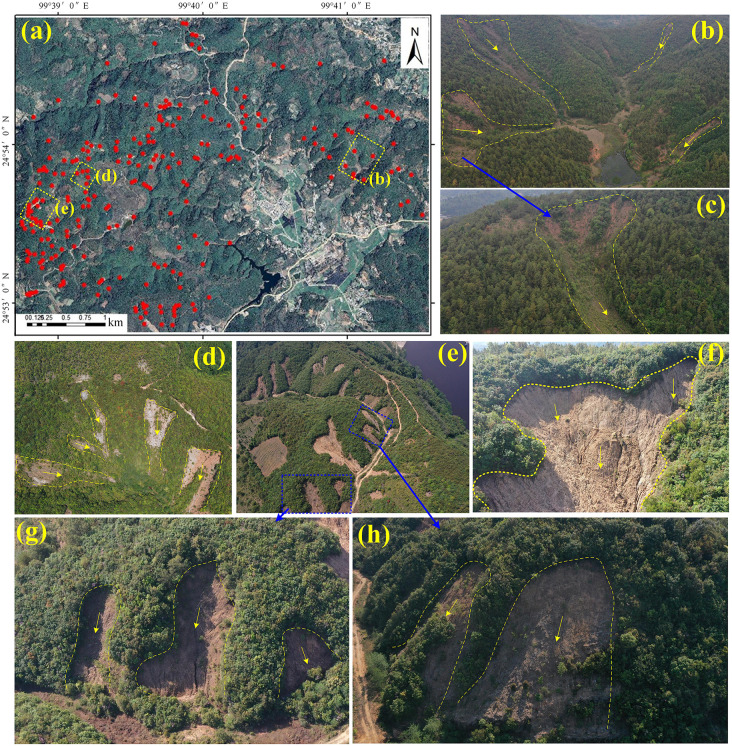
Landslide distribution in the study area. **(a)** Spatial distribution of landslides in the study area (red dots); (b, d, **e)** Three representative clustered landslide groups; (c, f, g, **h)** Field photographs showing the morphology of individual landslides.

On 16 September 2015, the county experienced an exceptional rainfall event, with a cumulative precipitation of 259.8 mm recorded between early morning and midday, triggering multiple landslides and debris flows, particularly affecting Tianyuan and Mangshui Towns, and resulting in 8 fatalities and 18 injuries. The landslide deposits accumulated within the gullies, creating large volumes of loose material that served as readily mobilizable sources for subsequent debris-flow activity. During the summer and autumn of 2016, sustained heavy rainfall again triggered numerous shallow, clustered landslides east of the Hexi Reservoir in Mangshui Town, further exacerbating the cascading geomorphic hazards in the area. Fig 2 illustrates the spatial distribution and typical morphological characteristics of rainfall-triggered landslides in the study area. Fig 2a presents the overall landslide distribution, with red dots indicating individual landslides, revealing their widespread occurrence; Fig 2b, 2d, 2e document three representative landslide clusters, highlighting their clustered and concentrated nature; Fig 2c, 2f–2h show several individual landslides, reflecting their scale, affected area, and environmental context. These events not only caused ecological degradation but also posed severe threats to the safety and property of local communities. Against this background, the present study focuses on two large-scale landslide clusters induced by extreme rainfall. Landslides were manually interpreted using high-resolution Google Earth imagery and supplemented with systematic field surveys to investigate their spatial distribution patterns and geological environment. The study aims to elucidate the spatiotemporal characteristics and predisposing conditions of rainfall-triggered landslides, providing a scientific basis for a deeper understanding of their causal mechanisms and evolution in southwestern Yunnan.

### 2.2. Data and methods.

This study draws upon a comprehensive dataset encompassing remote sensing imagery, digital elevation data, precipitation records, and geological information (Table 1). Remote sensing data primarily include Landsat 8/9 OLI/TIRS C2 L2 products obtained from the Geospatial Data Cloud, with panchromatic, multispectral, and thermal infrared resolutions of 15 m, 30 m, and 100 m (resampled to 30 m to maintain consistency with the multispectral data), covering 2013–present (Landsat 8) and 2021–present (Landsat 9). High-resolution imagery from 2015, 2016, and 2017 was additionally used to support multi-temporal visual comparison. These multi-source images provided the basis for manually delineating landslide polygons, which were subsequently validated through field investigations to ensure accurate landslide positioning and to offer a reliable foundation for the spatial analyses conducted in this study. Topographic attributes, including slope and aspect, were extracted from ASTER GDEM V3 (30 m). Monthly precipitation data (1 km, 1901 ~ 2023) were used to characterize rainfall patterns and assess landslide-triggering conditions. Geological background information, sourced from the 1:200,000 national geological map, provided lithological, stratigraphic, and fault data critical for evaluating lithological controls on landslide occurrence. Historical imagery from Google Earth (208 scenes, 15 ~ 30 m resolution) was employed for manual validation of landslide mapping. Road network data from Tianditu were used to examine spatial relationships between landslides and infrastructure. Landslide spatial distribution was analyzed in ArcGIS 10.8 and ENVI 5.6, systematically quantifying six controlling factors: elevation, slope, aspect, precipitation, normalized difference vegetation index (NDVI), and lithology.

**Table 1:** Data sources used in this study.

Data type	Source	Spatial resolution	Temporal resolution / coverage	Data content	Application
Landsat 8 OLI_TIRS satellite digital products	Geospatial Data Cloud Platform	Panchromatic: 15 m; Multispectral: 30 m; Thermal infrared: 100 m (resampled to 30 m)		Visible, NIR, SWIR, and TIR bands	Landslide recognition, land-cover classification, vegetation index analysis
ASTER GDEM 30M resolution digital elevation data	Geospatial Data Cloud Platform	30 m	—	Global digital elevation model	Extraction of slope, aspect, and other topographic factors
Geological map of China (1:200,000)	China Geological Survey	—	—	Lithology, stratigraphy, faults, and tectonic structures	Geological background and lithological control analysis
Google Earth historical imagery (208 scenes)	Google Earth	~15 ~ 30 m (depending on year/sensor)	Past ~30 years	High-resolution optical imagery	Landslide visual interpretation validation
Road network data	Tianditu, GS(2023) No.336		2023	Road distribution data	Analysis of spatial relationship between landslides and road networks

**Fig 3:**
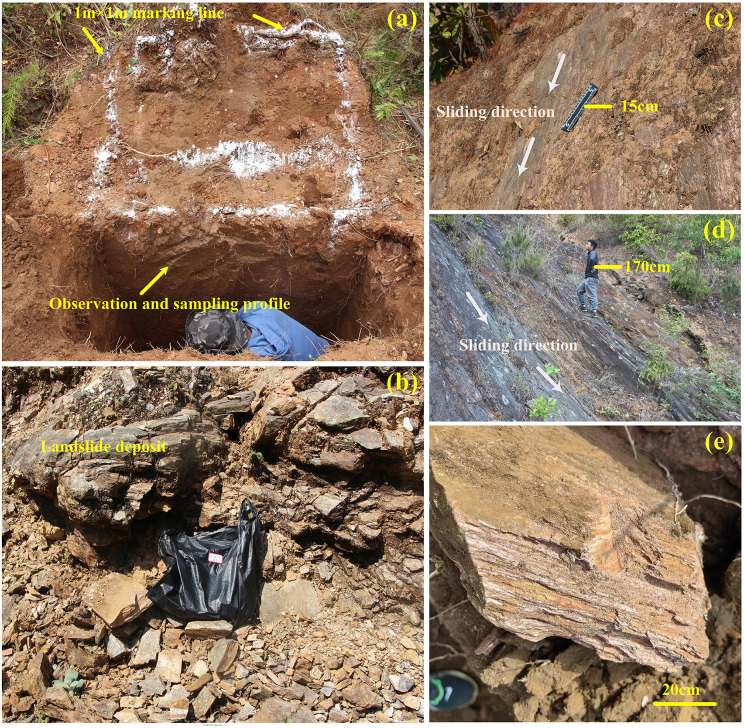
Field survey and sampling. (a) Soil sampling from the excavation at the landslide source area shown in Fig 2e; **(b)** Landslide deposits corresponding to Fig 2h; **(c)** Slip surface of the landslide in Fig 2e; **(d)** Slip surface of the landslide in Fig 2b; **(e)** Strongly weathered schist within the landslide deposit in Fig 2e.

To investigate the microscale characteristics of landslide materials, samples were collected from both landslide deposits and slip surfaces (Fig 3b–e). Sample preparation followed Liu et al [38]: intact soil samples were air-dried and impregnated with AB epoxy resin at a 3:1 ratio to fill pore spaces and consolidate the material, degassed using a SIE-400Z vacuum pump (SIENOX, China), and left to cure for three days. Thin sections (0.03 mm) were prepared using a Brillant220 cutter and Saphir 550 polisher (ATM, Germany). Optical observations were conducted with a 59XC-PC polarizing microscope (Shanghai Optical Instrument Factory, China) to characterize microstructural features and identify mineralogical components. Scanning electron microscopy (SEM) using a TESCAN MIRA LMS provided high-resolution surface morphology observations.

To assess the mechanical properties of the landslide source soils, samples were collected at 20 cm vertical intervals from an excavation at the landslide source area (Fig 3a) [39]. Direct shear tests were conducted under four normal stresses (50, 100, 200, and 300 kPa), with continuous recording of shear stress–shear strain to generate full stress–strain curves. Peak shear stress at failure was extracted from each curve and used to quantify shear strength under the corresponding normal stress.

## 3. Results and analysis

### 3.1. Landslide spatial distribution

#### 3.1.1. Topography.

**Fig 4:**
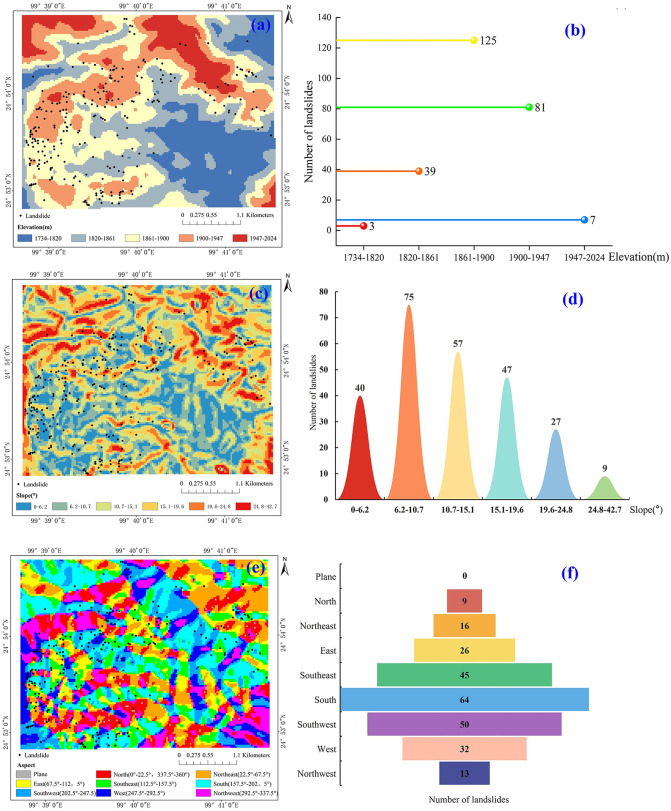
Spatial distribution and topographic controls of landslides: (a) landslides relative to elevation; (b) landslide counts across elevation classes; (c) landslides relative to slope angle; (d) landslide counts across slope-angle classes; (e) landslides relative to slope aspect; (f) landslide counts across slope-aspect categories.

Through manual interpretation of high-resolution Google Earth imagery, we identified a total of 255 landslides. To further clarify how topographic factors control their spatial distribution, we performed spatial statistical analyses in terms of elevation, slope gradient, and aspect. Elevation within the study area (1734 ~ 2024 m) was classified into five intervals using the natural breaks method. Landslide occurrences display a distinct unimodal pattern (Fig 4a, b). Among the 255 identified landslides, only 3 (1.18%) fall within the 1734 ~ 1820 m interval, representing the sparsest zone; 39 landslides (15.29%) occur in the 1820 ~ 1861 m range, showing a stepwise increase; the 1861 ~ 1900 m interval represents the peak concentration with 125 landslides (49.02%), forming the core elevation band in terms of both number and density. This elevation range likely corresponds to zones of enhanced weathering, moderate slope gradients, and stronger hydrological convergence, which commonly coincide with concave and convergent mid-slope geometries that further concentrate subsurface flow. The 1900 ~ 1947 m interval contains 81 landslides (31.76%), and the highest elevation interval, 1947 ~ 2024 m, hosts only 7 landslides (2.75%). In general, the elevation pattern can be described as “sparse at low elevations, dense at mid~high elevations, moderate at mid~upper elevations and sparse at the highest elevations,” with the 1861 ~ 1900 m clearly representing the dominant elevation range. The lowest and highest elevation intervals together account for less than 4% of landslides, highlighting the strong regulatory effect of elevation on landslide spatial distribution.

Slope gradient similarly exerts a critical control over landslide initiation. As slope increases, the tangential component of gravity along the slope rises while the normal component diminishes, promoting mechanical instability in slopes around 20° ~ 40° (Fig 4c, d). Statistical analysis indicates that most landslides occur on moderate slopes: 6.2° ~ 10.7° hosts 57 landslides, 15.1° ~ 19.6° contains 54, and the remaining intervals 0° ~ 6.2° and 10.7° ~ 15.1° each contain 51 landslides. Slopes above 19.6° show sharply reduced occurrences, with only 28 and 14 landslides in the 19.6° ~ 24.8° and 24.8° ~ 42.7° intervals, respectively. These data indicate a non-linear relationship between slope and landslide occurrence, suggesting a threshold effect near 20° as the critical range for slope instability.

Slope aspect regulates solar radiation, rainfall distribution, soil moisture, and vegetation cover, indirectly shaping landslide spatial patterns. The study area was classified into nine aspect categories: flat, N, NE, E, SE, S, SW, W, and NW. South-facing slopes host the highest number of landslides (64), followed by southeast-facing slopes (45), together accounting for 42.7% of all landslides; southwest-facing slopes contain 50 landslides (19.6%) (Fig 4e, f). Collectively, south- and southeast-facing slopes account for 62.3% of total occurrences, reflecting pronounced directional clustering. This pattern likely results from enhanced solar radiation and rainfall exposure on these windward slopes, accelerating physical and chemical weathering, which enhances soil particle disaggregation, increases pore-water content, and reduces cohesion and internal friction angle, thereby lowering slope shear strength and increasing landslide susceptibility. Conversely, flat and north-facing slopes exhibit minimal landslide (0 and 9 occurrences, respectively), likely due to reduced solar irradiation, lower rainfall accumulation, and higher vegetation cover, which protect the slopes from failure. These results indicate that aspect exerts a strong directional control on landslide occurrence through the combined effects of radiation, precipitation, and vegetation, making sunlit and windward slopes particularly vulnerable.

#### 3.1.2. Vegetation conditions.

**Fig 5:**
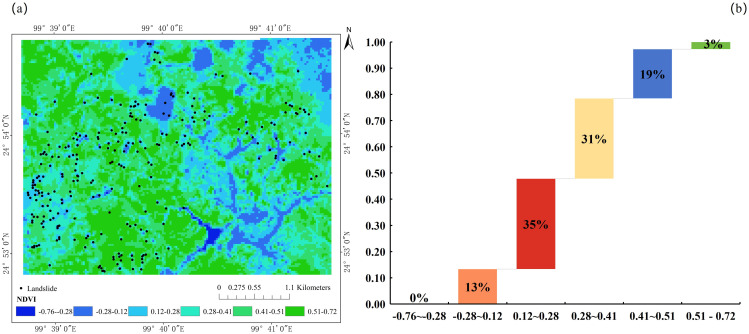
Landslide distribution and counts across NDVI classes: (a) spatial distribution of landslides relative to NDVI; (b) landslide counts by NDVI interval.

NDVI is a remote-sensing–derived metric widely used to quantify vegetation cover and vigor by exploiting the differential reflectance of vegetation in the red (Red) and near-infrared (NIR) spectral bands [40,41]. NDVI is computed as: NDVI=(NIR-Red)/ (NIR + Red), yielding values from –1–1, where higher values correspond to denser and more vigorous vegetation. NDVI has been widely used as a proxy for vegetation density and surface conditions in landslide-related studies, facilitating the analysis of relationships between vegetation cover and landslide spatial distribution [42,43]. Vegetation influences landslide occurrence through its effects on surface cover, root reinforcement, and hydrological regulation. As shown in Fig 5, landslides exhibit a distinct distribution pattern across NDVI classes. In areas with extremely low NDVI values, landslides are exceedingly rare. These zones typically correspond to densely populated settlements where site selection and engineering protection measures (e.g., slope stabilization and drainage) effectively suppress landslide occurrence. This highlights the coupled influence of NDVI and human activities on the spatial pattern of landslides.

The NDVI interval of 0.12 ~ 0.28 hosts the largest proportion of landslides (35%), with 89 events occurring within this range. These areas are characterized by sparse vegetation or exposed surfaces where soils are highly susceptible to rainfall-driven erosion and weathering. Even during the rainy season, moderate increases in NDVI do not substantially mitigate instability, as elevated soil moisture and insufficiently developed root systems maintain a high failure potential. Anthropogenic disturbances such as deforestation and road construction further reduce NDVI, particularly on steep slopes, thereby amplifying landslide susceptibility. Conversely, high-NDVI zones (0.41 ~ 0.51) display very low landslide frequencies (3%, or only 8 events). Field surveys indicate that the dominant vegetation in these areas includes *Pinus yunnanensis*, *Cunninghamia lanceolata*, and *Buddleja officinalis*, which are interspersed across the landslide blocks. Dense vegetation with well-developed root networks in these zones substantially enhances soil cohesion and slope stability, thereby reducing landslide likelihood.

#### 3.1.3. Distance to roads.

**Fig 6:**
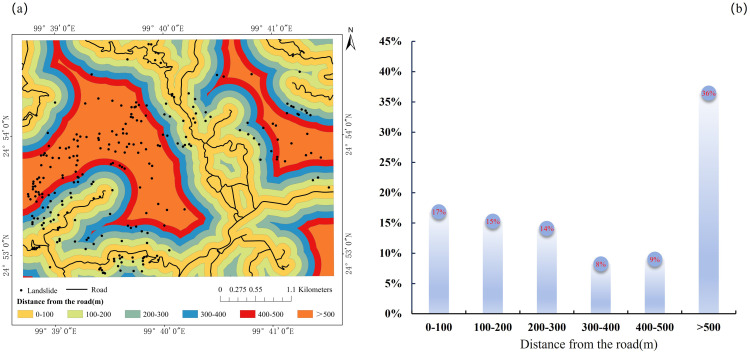
Landslide distribution and counts relative to road proximity: (a) spatial distribution of landslides relative to roads; (b) landslide counts across distance-to-road intervals.

Road construction is widely recognized as a potential modifier of slope stability, both directly through terrain excavation and loading, and indirectly through hydrological disturbance and vegetation removal. Consequently, numerous large-scale studies have reported elevated landslide densities adjacent to road networks, identifying road distance as a significant linear predictor of landslide spatial patterns [44]. However, the effect of roads is not universally monotonic and must be interpreted within the context of intrinsic slope conditions and the scale of engineering disturbance. While excavation and embankment loading can locally alter stress states and increase failure susceptibility, road alignment practices often avoid steep gradients and mechanically weak lithologies, thereby reducing their direct association with slope instability. The statistical distribution of landslides across distance-to-road classes (Fig 6) shows the following pattern: 0 ~ 100 m (17%), 100 ~ 200 m (15%), 200 ~ 300 m (14%), 300 ~ 400 m (8%), 400 ~ 500 m (9%), and >500 m (36%). The highest proportion of landslides occurs beyond 500 m, substantially exceeding that of road-proximal zones. This pattern does not align with the commonly observed concentration of failures near engineered slopes. Instead, it indicates that in this region, landslide occurrence exhibits only a weak spatial association with the road network. A key explanation is that the study area is predominantly served by low-grade rural roads with limited excavation depth and minimal anthropogenic alteration of slope geometry or hydrogeological conditions. Field observations corroborate that engineering disturbance is negligible compared to the inherent geological and geomorphological predispositions of the slopes. Overall, the spatial distribution of landslides suggests that human activities, particularly road construction, play a relatively minor role in controlling slope failures in this setting. Instead, landslide occurrence is primarily governed by natural factors, including lithological structure, slope gradient, degree of weathering, and the strong infiltration-driven weakening associated with extreme rainfall events.

#### 3.1.4. Lithology.

**Fig 7:**
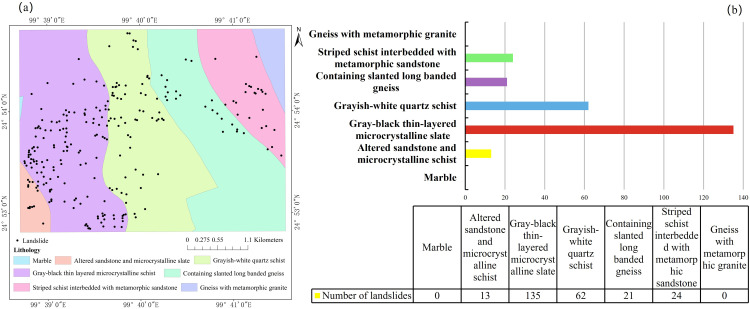
Landslide distribution and frequency relative to lithology: (a) spatial distribution of landslides across lithological units; (b) landslide occurrence by lithology.

The distribution of landslides is closely tied to lithology, which constitutes one of the most fundamental geological controls on slope susceptibility. Variations in mineral composition, mechanical strength, weathering properties, and structural fabrics lead to pronounced differences in landslide frequency across lithological units [45]. Integration of mapped landslide locations with the national geological dataset shows substantial contrasts among lithologies (Fig 7). Grey-black thin-bedded microcrystalline schist hosts the highest number of landslides (135), followed by grey-white quartz schist (62). Fewer landslides occur in banded schist interlayered with metasandstone (24), plagioclase-bearing metaporphyroblastic schist (21), and metasandstone– microcrystalline schist units (13). No landslides were recorded within Carboniferous marble or Paleozoic gneiss interlayered with metagranulite, suggesting that these competent and relatively massive rock units possess high mechanical strength, superior resistance to weathering, and robust shear strength,attributes conducive to long-term slope stability.

On the whole, the pronounced clustering of landslides within schistose formations reflects well-recognized relationships between schistosity and slope instability. Due to their foliated fabrics, anisotropy, and preferential weathering along structural planes, schists are widely documented as high-susceptibility lithologies [46,47], and numerous studies have reported elevated landslide densities in schist-dominated terrains [48].

### 3.2. Analysis of landslide-forming mechanisms

#### 3.2.1. Macro- and micro-structural characteristics of the rock mass.

**Fig 8:**
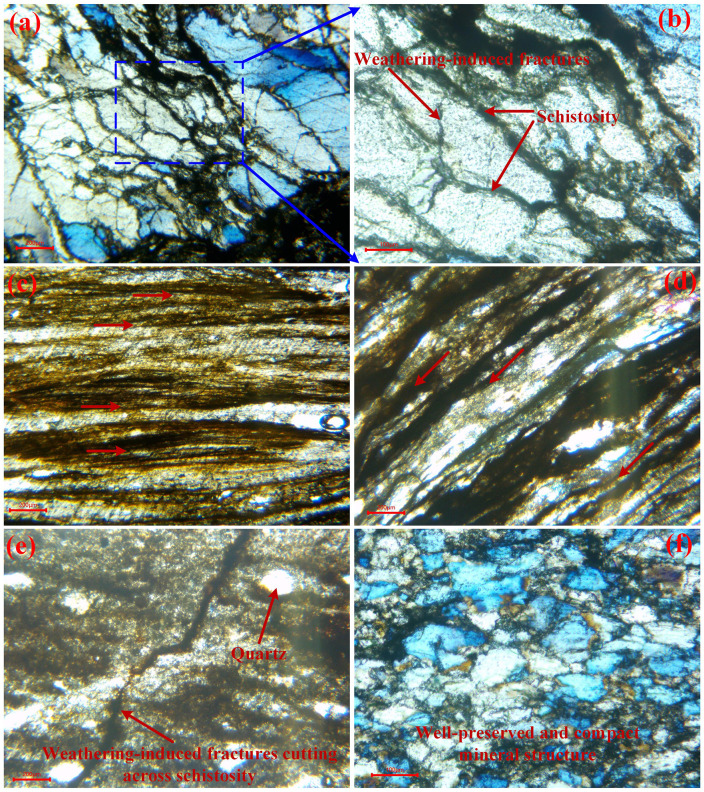
Polarized-light microstructures of schist bedrock under different degrees of weathering. (a ~ b) Extensive foliation disrupted by pervasive weathering-induced fractures; (c ~ d) sharply defined, continuous foliation characteristic of the primary metamorphic fabric; (e) large weathering fissures truncating mineral banding; (f) compact texture with well-preserved primary mineral assemblages.

Polarized-light microscopy shows that regional metamorphism combined with sustained directional stress has driven a strong crystallographic reorientation of quartz, mica, and associated minerals, producing well-defined, subparallel foliation and banded fabrics. These planar structures are locally crosscut by weathering-induced fractures that disrupt lithologic continuity and diminish rock-mass integrity (Fig 8a–b). Dark staining and banded features, dominated by limonite, hematite, and other iron oxides, are preferentially distributed along foliation or fracture surfaces, reflecting oxidation fronts developed during advanced weathering. Fine-scale microcracks are also present; many are partially or fully filled with secondary alteration products, further underscoring the progressive breakdown of the rock’s internal cohesion (Fig 8e). Alternating light and dark mineral layers (Fig 8c–d) typify the schistose texture. Mica group minerals, occurring in platy and flaky habits, are strongly aligned parallel to foliation and constitute the primary structural signature of the schist. The lighter bands are dominated by quartz with subordinate relict feldspar grains, typically transparent to semi-translucent under polarized light. This prominent layering, together with the pervasive foliation, imposes strong mechanical anisotropy and substantially weakens the rock’s shear resistance. Mineralogical contrasts further accentuate this behavior: quartz and plagioclase represent brittle, high-strength phases with relatively robust resistance to weathering, whereas biotite and chlorite behave as weak, hydrophilic phyllosilicates that soften markedly upon hydration [49]. Their preferential alignment along foliation makes the rock mass especially prone to splitting, delamination, and shear localization. Less-weathered specimens retain largely intact mineral components (Fig 8f), consisting primarily of quartz and feldspar with minor chlorite, biotite, and iron-rich minerals. Although individual grains remain morphologically preserved, they are predominantly fine- to very fine-grained with blurred grain boundaries, yielding a dense to moderately loosened weathering fabric. Early-stage weathering induces subtle mineralogical changes, but progressive alteration increases porosity, microcracking, and intergranular weakness.

Taken together, the schists in the Changning region display a characteristic metamorphic foliation inherited from regional tectonism, yet prolonged weathering profoundly modifies their internal structure. Weathering-generated fractures interrupt foliation continuity, magnify heterogeneity and anisotropy, and sharply undermine rock-mass integrity. These coupled mineralogical and structural evolutions substantially reduce the mechanical competence of the schist on slopes, rendering it a highly failure-prone substrate and forming a critical geological foundation for rainfall-induced landslides in the area.

**Fig 9:**
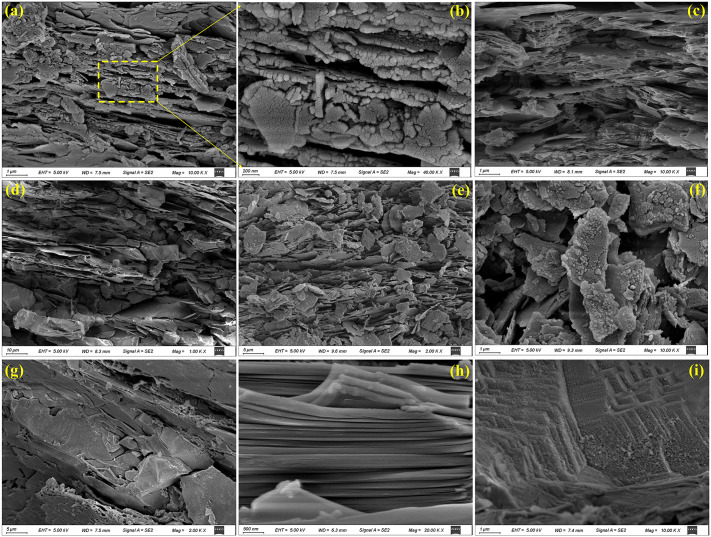
SEM microstructural characteristics of schist at various weathering stages. (a ~ c) strongly weathered schist from the landslide site in Fig 2c, showing degraded foliation and disaggregated mineral fabrics; (d ~ f) strongly weathered schist from the site in Fig 2f, exhibiting intense microfracturing and mineral breakdown; (g ~ i) weakly weathered schist from the landslide in Fig 3d, retaining a relatively dense and coherent foliated structure.

**Fig 10:**
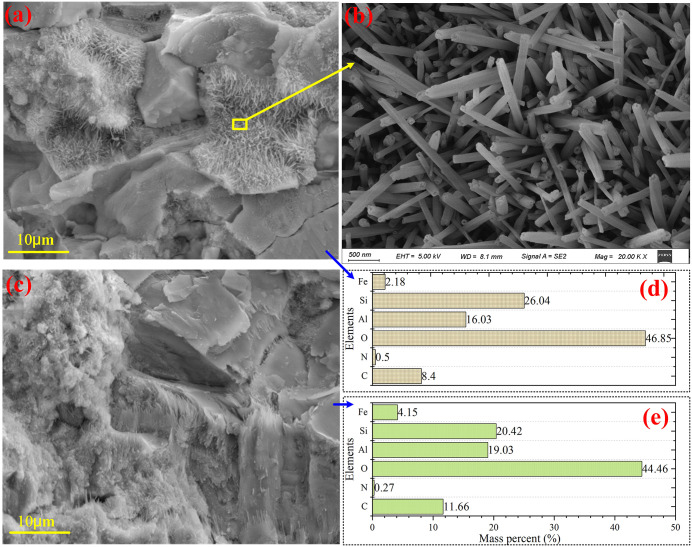
Elemental signatures of intensely weathered schist. (a ~ b) SEM images of highly altered microzones showing secondary mineral infilling and disrupted fabric; (c) elemental composition corresponding to the region delineated in **(a)**; (d) elemental composition corresponding to the region delineated in **(b)**.

Scanning Electron Microscopy (SEM) of the schist reveals that its foliation remains distinctly identifiable at the microscale. This structural fabric reflects the preferential alignment of mineral grains, particularly platy and lamellar mica minerals, formed under regional metamorphism and persistent directional stress (Fig 9a–c). With progressive weathering, however, portions of the foliation become disrupted, and mineral grains appear more dispersed, producing a loose and irregular microtexture (Fig 9e–f). Energy-dispersive spectral analysis confirms abundant secondary minerals, dominated by aluminosilicates (Fig 10), indicative of intense mineral alteration and elemental migration.The disaggregated distribution of platy minerals highlights their pronounced cleavage and strong structural anisotropy, which collectively reduce the compressive strength of the rock mass. Under advanced weathering, partial breakdown of mineral lattices becomes evident; some grains exhibit increased brittleness, bending, or deformation under stress (Fig 9d). Weathering also promotes the formation of microcracks and intergranular pores, accelerating fluid migration and enhancing porosity, thereby further diminishing the density and mechanical stability of the rock mass. In contrast, weakly weathered schist retains a relatively dense fabric, with well-preserved foliation and closely packed mineral aggregates. Minor surface warping or bending is locally observed (Fig 9g–i), suggesting latent susceptibility to cleavage-controlled rupture. Overall, weathering drives a progressive transition from a dense, well-foliated structure to a loose, porous, and mechanically weakened fabric.

**Fig 11:**
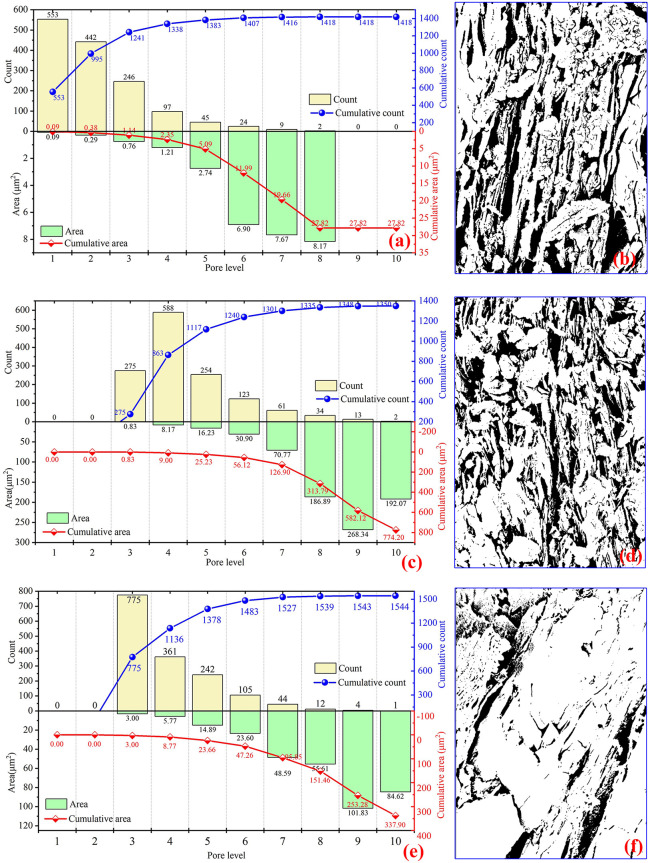
Pore characteristics of schist under different weathering intensities. **(a)** Pore number-area extracted from the microstructure of Fig 9a; (b) binarized representation of Fig 9a; (c) pore number-area extracted from Fig 9e; (d) binarized representation of Fig 9e; (e) pore number-area extracted from Fig 9g; (f) binarized representation of Fig 9g.

To quantitatively elucidate the influence of weathering on the structural evolution of schist, SEM imaging and digital pore-structure analyses were conducted on samples collected from several representative landslide source zones (Fig 9a, 9e, 9g). The original micrographs were binarized (Fig 11b, 11d, 11f) to extract pore geometries and quantify pore-size distributions (Fig 11a, 11c, 11e). Measured pore diameters range from 0.0122 μm to 12.2 μm. To accommodate the abundance of nanoscale pores while limiting the dominance of large voids in the overall statistics, pore sizes were grouped logarithmically into ten levels, ranging from 0.010 ~ 0.021 μm (pore level 1) to 7.91 ~ 20.0 μm (pore level 10). The resulting pore-number distributions reveal striking contrasts between weakly and strongly weathered schist. Weakly weathered samples contain a larger total pore count (1,544) than strongly weathered samples (1,350). Fine pores, especially those within levels 2 ~ 3, dominate the weak-weathering assemblage, reflecting matrix-scale microporosity and narrow foliation-parallel voids. Coarse pores (levels ≥7) are scarce, with only 61 identified. In strongly weathered schist, however, although micropores still constitute the numerical majority (1,241 pores within levels ≤3), the number of large pores increases substantially, reaching 110 at levels ≥7, indicating pervasive fracture widening and the development of weathering-induced macrovoids. Pore-area statistics further amplify these contrasts. The total pore area in the weakly weathered schist is 337.90 μm², showing a gradual increase with pore size and only limited contributions from coarse pores. In comparison, the strongly weathered schist exhibits a total pore area of 774.20 μm², more than twice that of the weakly weathered samples, with exceptionally rapid growth in levels 8 ~ 10. Although fewer in number, these large pores dominate the total area, reflecting intense mineral decomposition, fracture coalescence, and mass transfer during advanced stages of weathering.

Existing quantitative comparisons between microstructural parameters obtained from scanning electron microscopy (SEM) and macroscopic mechanical behavior demonstrate that weathering significantly enlarges pores and microcracks within schist, resulting in a pronounced nonlinear decay of uniaxial compressive strength (UCS) with increasing porosity. Previous studies indicate that, from fresh to completely weathered stages, porosity may increase by up to 158-fold, and every 10% increase in porosity leads to an average 60 ~ 70% reduction in UCS, with strength decreasing to as low as 2% of its original value [50]. This confirms a strong negative correlation between rock strength and weathering degree. Further quantitative relationships show that UCS (σc) exhibits an exponential decay with both effective porosity (ne) and total porosity (nt), following σc = 125e^−0.20ne^ and σc = 195e^−0.21nt^, respectively [51,52]. Meanwhile, weathering-induced crack propagation increases rock mass permeability by more than an order of magnitude.

These micro-scale degradation processes not only cause substantial strength reduction but also accelerate rainfall infiltration and promote rapid pore-water pressure buildup, thereby facilitating preferential shear slip along foliation planes. The weakening effect is particularly pronounced where foliation dip direction is nearly parallel to the slope aspect, providing a direct explanation for the observed spatial clustering of landslides at the field scale. Overall, this process offers critical micro- to macro-scale evidence for understanding the failure mechanisms of rainfall-induced landslides in schist-dominated terrains.

#### 3.2.2. Mechanisms of landslide triggering under rainfall conditions.

Rainfall is widely recognized as the primary external trigger for landslide initiation. The study area experienced an extreme precipitation event from 02:00 on 16 September to 23:00 on 17 September 2015. In Mangshui Town, intense rainfall began at 02:00 on 16 September, with an 8-hour cumulative rainfall of 227.8 mm from 02:00–10:00, a total daily precipitation of 239.6 mm, and hourly rainfall rates ranging from 19.2 to 44.8 mm. At Hexi Reservoir station in Tianyuan Town, the 8-hour cumulative rainfall from 02:00–10:00 reached 252.9 mm, with a daily total of 263.3 mm,the highest on record for Changning County. From 03:00 onwards, rainfall intensified, with hourly rates between 8.2 and 108.9 mm; five hourly intervals exceeded 15 mm, and a peak of 108.9 mm occurred within a single hour between 08:00 and 09:00, representing an extreme intensity. This event thus constituted a prolonged, high-intensity rainfall episode. In 2016, precipitation in Changning County was concentrated from May to October, accounting for nearly 80% of the annual total, with the highest rainfall recorded in August at 247 mm [53,54]. Such sustained heavy rainfall events altered the physical and mechanical properties of the schist bedrock and overlying weathered slope deposits, thereby creating favorable conditions for landslide initiation.

**Fig 12:**
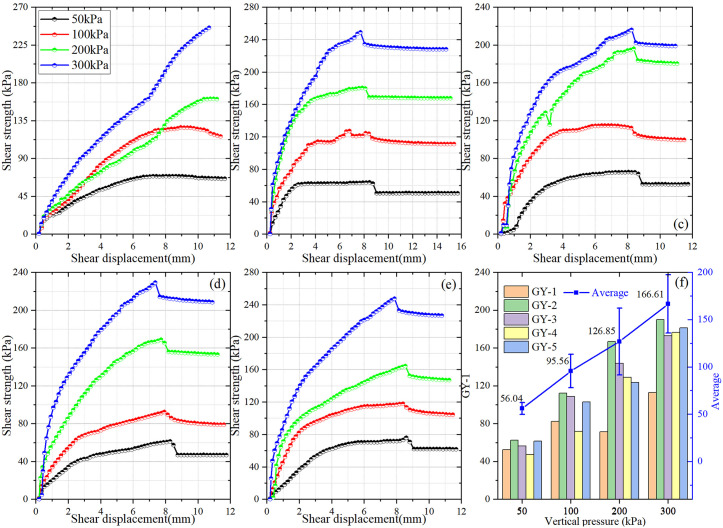
Direct shear test results for source-area soils from the clustered landslides in Changning. (a ~ e) Shear stress– displacement responses for soil samples GY1–GY5 excavated from the source zone shown in Fig 3e;(f) derived shear-strength parameters, highlighting contrasts in peak and residual strength among samples.

To quantify the mechanical response of source-area soils, samples were collected at various depths (Fig 3a) and subjected to direct shear tests. Shear stress–displacement curves (Fig 12a–e) exhibit typical behavior: shear stress rises with increasing displacement and gradually stabilizes, with peak stress values increasing approximately linearly with normal stress. Peak shear displacements typically range from 6.7 to 10.8 mm, with minor increases at higher normal stresses. At elevated normal stresses, the curves steepen, peak stresses occur at larger displacements, and post-peak degradation is limited, occasionally approaching a plateau indicative of residual shear capacity. In contrast, under lower normal stresses, the difference between peak and residual strength is pronounced, demonstrating clear strain-softening behavior. Derived shear-strength parameters (Fig 12f) indicate a strong positive dependence on normal stress, with mean shear strength increasing from 56.04 kPa at 50 kPa normal stress to 166.61 kPa at 300 kPa. Layer-specific differences are evident: sample GY1 exhibits substantially lower shear strength than other layers at identical normal stresses, reflecting a relatively loose structure with limited resistance to shear failure, whereas GY2, GY3, and GY5 display higher strengths at medium-to-high stresses, indicative of denser, mechanically stable soils.

Integrating these mechanical insights with regional geological and environmental characteristics highlights the critical role of rainfall in reducing the shear strength of fully weathered schist. Infiltrating water increases soil moisture and pore-water pressures, diminishing effective normal stress and directly reducing key shear-strength parameters, including cohesion *(c)* and internal friction angle *(φ)*. Previous studies suggest that, for highly weathered rocks, saturation can reduce *φ* by 20–40% relative to dry conditions and decrease cohesion by more than 50%. When saturation exceeds 60%, shear strength declines sharply, and near-saturation conditions can dramatically compromise slope resistance [55]. In highly fractured, porous schist, water more readily penetrates and accumulates, accelerating pore-pressure buildup and strength degradation. Water infiltration also disrupts particle cementation and adsorption, further reducing effective cohesion, while lubrication and decreased particle contact lower the internal friction angle. The abundant foliation, joints, and faults characteristic of schist become mechanically weakened under rainfall, reducing friction along these potential slip surfaces and facilitating the development of failure planes. In summary, rainfall acts as a decisive triggering factor for landslide initiation by altering the hydro-mechanical state of the rock mass and weakening the structural integrity and shear strength of fully weathered schist.

## 4. Discussion

This study investigates the spatial distribution patterns and controlling mechanisms of clustered shallow landslides in a high-mountain canyon environment underlain by metamorphic rocks. This study uses landslide clusters as the primary analytical unit. It integrates geological, topographic, vegetation, and microstructural analyses to reveal the role of multiple interacting factors and cross-scale interactions governing landslide occurrence. The results indicate that slope failures in schist-dominated mountainous regions are governed by the interaction of multiple factors rather than a single triggering process [56]. The clustered landslides in Changning County clearly exhibit such multi-factor coupling, reflecting the combined influence of geological structures, lithological anisotropy, weathering evolution, geomorphological configuration, and external hydroclimatic forcing [57]. This finding extends previous studies that primarily focus on single landslide events or site-specific triggering mechanisms [58,59], by incorporating broader lithological controls, structural continuity, and spatial clustering into the analysis. Although such studies provide valuable insights into local processes, they may not fully capture the spatial organization of failure clusters and the role of large-scale structural continuity.

In the Changning area, prolonged tectonic deformation and sustained weathering have produced pervasive foliation and dense fracture networks within the schist. These anisotropic discontinuities act both as mechanical weak planes and as preferential flow paths that facilitate rapid rainfall infiltration, localized pore-pressure buildup, and stress redistribution. Once infiltration becomes concentrated along structurally weakened zones, even relatively small hydrological perturbations may escalate into large-volume slope failures [7,10,11]. The observed landslide behavior reflects a hierarchical coupling across multiple spatial scales. At the regional and slope scale, tectonic architecture and lithological contrasts govern the spatial distribution and geometry of potential failure units. At intermediate scales, the thickness of weathering profiles and spatial variability in material properties influence hydraulic transmissivity, water retention, and the rate of shear strength degradation under wetting conditions. At the microstructural scale, pore size distribution and microfracture connectivity directly affect permeability, softening behavior, and effective stress evolution [13,14,17,49]. Interactions across these scales may produce nonlinear amplification effects, consistent with previous studies highlighting the tight coupling between hydrological and geomechanical processes in slope instability [57–59]. These results emphasize the importance of considering cross-scale interactions when analyzing landslide spatial patterns in structurally complex metamorphic terrains.

The findings of this study also have broader implications for landslide hazard assessment in metamorphic regions across Yunnan Province. Schist is widely distributed due to regional tectonic and metamorphic evolution, forming spatially clustered outcrops in northwestern, southwestern, and parts of central Yunnan. These terrains, characterized by strong foliation, abundant discontinuities, and deep weathering, are particularly susceptible to clustered slope failures under the combined influence of extreme rainfall and material weakening. Improving hazard assessment in such environments requires a more comprehensive understanding of the interactions among tectonics, lithology, weathering, and hydrological processes. From an application perspective, advancing landslide hazard assessment will benefit from integrated, multi-source geospatial datasets that include geological structures, lithological distributions, weathering profiles, meteorological records, and hydrological conditions [17,25,60]. Multi-temporal optical imagery and InSAR techniques can monitor long-term slope deformation, while data integration approaches may help identify areas with strong coupling between rainfall infiltration and mechanical weakening [61]. High-risk slopes may also require dense monitoring systems, including rainfall gauges, pore-pressure sensors, inclinometers, GNSS stations, and UAV-based topographic observations, to better constrain coupled hydro-mechanical processes [60]. Building on these integrated observations and monitoring efforts, developing multi-factor predictive frameworks tailored to metamorphic terrains is essential for identifying critical spatiotemporal thresholds and improving early warning capabilities for clustered landslides [60,62]. At the same time, this study has some limitations, as the relative quantitative contributions of individual controlling factors were not explicitly assessed. Future research should focus on quantifying these contributions to further refine understanding of landslide triggering mechanisms.

## 5. Conclusions

(1) A total of 255 landslides were identified within the study area, forming belt-like clusters along major valleys, with their spatial variability governed jointly by elevation, slope angle, slope aspect, and lithology. Failures predominantly occur at mid-to-high elevations and on moderate slopes, with south- and southeast-facing slopes being most susceptible. Gray–black thin-layered microcrystalline schist and light gray quartz schist represent the primary lithological units associated with failures, while the influence of road construction is comparatively minor, as natural slopes beyond 500 m from roads show higher landslide densities, underscoring the dominant role of terrain and lithological controls.
(2) Microstructural analyses reveal that schist within the landslide zones exhibits pervasive foliation, cleavage, and weathering-induced fracturing, with weathering driving a marked transition from dense to porous structures. Weakly weathered schist shows pore structures dominated by small pores, whereas strongly weathered schist exhibits significantly greater total pore area and a higher abundance of large pores and microfractures. This evolution toward greater porosity and fracture connectivity weakens shear resistance, enhances permeability, and establishes the microstructural basis for slope instability.
(3) Direct shear testing indicates that peak shear strength increases with normal stress, yet shallow and loose samples consistently display lower shear resistance. Intense, short-duration rainfall acts as the primary trigger of widespread slope failures. Rapid increases in soil moisture and pore-water pressure reduce effective stress, weaken shear strength, and activate preexisting weak planes. Localized infiltration can therefore escalate into large-scale, clustered failures once structural and hydrological thresholds are exceeded. The results collectively demonstrate that rainfall-induced alterations in pore-water dynamics, combined with weathering-controlled mechanical degradation, constitute the key mechanisms driving landslide initiation in schist-dominated terrains.
